# A combined preoperative red cell distribution width and carcinoembryonic antigen score contribute to prognosis prediction in stage I lung adenocarcinoma

**DOI:** 10.1186/s12957-023-02945-7

**Published:** 2023-02-22

**Authors:** Hengliang Xu, Guangqiang Zhao, Jixing Lin, Qianwen Ye, Jia Xiang, Bing Yan

**Affiliations:** 1 Department of Thoracic Surgery, Hainan Hospital of Chinese PLA General Hospital, Sanya, Hainan 572000 People’s Republic of China; 2 Department of Respiratory Medicine, Sanya Peoples’ Hospital, Sanya, Hainan 572000 People’s Republic of China; 3 Department of Oncology, Hainan Hospital of Chinese PLA General Hospital, No. 80 of Jianglin Road, Haitang District, Sanya, Hainan 572000 People’s Republic of China

## Abstract

**Aims:**

Hematological markers that can be used for prognosis prediction for stage I lung adenocarcinoma (LUAD) are still lacking. Here, we examined the prognostic value of a combination of the red cell distribution width (RDW) and carcinoembryonic antigen (CEA), namely, the RDW-CEA score (RCS), in stage I LUAD.

**Materials and methods:**

A retrospective study with 154 patients with stage I LUAD was conducted. Patients were divided into RCS 1 (decreased RDW and CEA), RCS 2 (decreased RDW and increased CEA, increased RDW and decreased CEA), and RCS 3 (increased RDW and CEA) subgroups based on the best optimal cutoff points of RDW and CEA for overall survival (OS). The differences in other clinicopathological parameters among RCS subgroups were calculated. Disease-free survival (DFS) and OS among these groups were determined by Kaplan–Meier analysis, and risk factors for outcome were calculated by a Cox proportional hazards model.

**Results:**

Seventy, 65, and 19 patients were assigned to the RCS 1, 2, and 3 subgroups, respectively. Patients ≥ 60 years (*P* < 0.001), male sex (*P* = 0.004), T_2_ stage (*P* = 0.004), and IB stage (*P* = 0.006) were more significant in the RCS 2 or 3 subgroups. The RCS had a good area under the curve (AUC) for predicting DFS (AUC = 0.81, *P* < 0.001) and OS (AUC = 0.93, *P* < 0.001). The DFS (log-rank = 33.26, *P* < 0.001) and OS (log-rank = 42.05, *P* < 0.001) were significantly different among RCS subgroups, with RCS 3 patients displaying the worst survival compared to RCS 1 or 2 patients. RCS 3 was also an independent risk factor for both DFS and OS.

**Conclusions:**

RCS is a useful prognostic indicator in stage I LUAD patients, and RCS 3 patients have poorer survival. However, randomized controlled trials are needed to validate our findings in the future.

## Introduction

Lung cancer is still the leading cause of cancer-related death worldwide, with an estimated 1.8 million deaths in 2020 [1]. Fortunately, with the increasing popularity of low-dose computed tomographic screening, the mortality of the disease has decreased remarkably in recent years, and patients can be treated at early stages [2, 3]. However, recurrence is frustratingly unavoidable, and the 5-year overall survival (OS) rates are 68–92% for stage I cases according to the eighth edition of the Tumor-Node-Metastasis (TNM) classification [4]. Reliable prognostic markers that can be used for these early patients are still needed.

Previously, except for the risk factors identified in the National Comprehensive Cancer Network guidelines for non-small cell lung cancer (NSCLC) [5] and pathologically micropapillary and solid patterns [6], tumor spread through air spaces [7] is regarded as an additional risk factor for stage I cases, and patients with these features are thought to be candidates to receive and benefit from adjuvant therapies (ADTs). However, hematological markers, which can be easily obtained from routine blood tests that could also guide the treatment for these patients, are still lacking. Red cell distribution width (RDW), which can reflect the size heterogeneity of circulating erythrocytes, was also found to be associated with inflammation, malnutrition, and impaired kidney function [8]. The prognostic value of RDW has been registered in many cancers according to a meta-analysis [9]. In lung cancer, Wang et al. conducted a meta-analysis and indicated that a higher value of pretreatment RDW was significantly associated with worse OS and disease-free survival (DFS) [10]; however, single RDW was less efficient in predicting the outcome since the reported area under the curve (AUC) ranged from 0.565 [11] to 0.629 [12, 13]. Additionally, these studies conventionally included stage I–IV cases, which may have greatly biased the results [11–13]. To date, only two studies have explored the prognostic value of RDW in stage I lung cancer [14, 15]; however, without reporting the definite AUC, more studies are still needed.

Carcinoembryonic antigen (CEA) is a tumor maker in NSCLC [16], and its prognostic value is under extensive study [17]. However, the positive rate of CEA is still limited (ranging from 19 to 33.6% [18–20]) in stage I cases, and its prognostic value is under debate in such a scenario. For example, Kuo et al. conducted a study with 758 stage I patients (541 adenocarcinomas (AD), 83 squamous cell carcinomas (SCC), and 134 others) and found that CEA was an independent risk factor for recurrence [21]. However, Blankenburg et al. conducted a study with 240 stage I NSCLC patients (91 AD, 100 SCC, and 32 others) and found that preoperative CEA could not be used to predict the 3- or 5-year OS with a cutoff value of 6.7 ng/mL [22]; in line with this, Maeda et al. performed a study that enrolled 229 stage IA patients (195 AD, 34 others) and suggested that preoperative CEA was not an independent risk factor for poor prognosis [23]. Nonetheless, it was notable that CEA was much more efficient in AD than other pathological phenotypes [24, 25] in NSCLC, and the majority of previous studies mixed with other pathological phenotypes could attenuate the prognostic efficacy of CEA. Interestingly, some authors have tried to combine hematological markers, such as platelets, with CEA to further improve its prognostic efficacy in NSCLC [26], and other studies have indicated that a combination of RDW with tumor markers would be useful for the prognosis of cancer patients [27, 28]. Based on these findings, we speculated that a combination of RDW and CEA could also be meaningful in the prognosis of stage I lung adenocarcinoma (LUAD); however, related reports are rare.

Here, we aimed to explore the prognostic value of a newly established indicator, namely, the combined RDW and CEA score (RCS), in stage I LUAD.

## Materials and methods

### Patients

From December 2012 to April 2019, data from patients who underwent surgery for lung cancer at Hainan Hospital of Chinese PLA General Hospital were retrospectively collected. Those who met any one of the following criteria were excluded: (1) suspected distant lesions by preoperative examinations, (2) any preoperative adjuvant therapies, (3) in situ lesions, (4) lack of preoperative laboratory tests, (5) lack of any pathological TNM (pTNM) information, (6) pTNM > I according to the 8th edition of AJCC [4], (7) pathological phenotypes other than AD, and (8) follow-up problems. Other parameters, including age, sex, type of resection, and others, were also recorded as in our previous reports [29, 30]. The study was performed in line with the principles stated in the Declaration of Helsinki and was supervised by the ethics committee of Hainan Hospital of Chinese PLA General Hospital. Written informed consent was waived due to its retrospective nature.

### Defining RCS and other inflammatory prognostic indicators

The data from routine blood tests were obtained within 1 week before curative surgery as previously described [29, 30]. CEA measurement was conducted in automated Cobas e601 immunoanalyzer (Roche Diagnostics GmbH, Germany) using an electrochemiluminescence immunoassay method with a reference at 0–5.0 ng/mL. Patients were divided into RDW-low or RDW-high and CEA-low or CEA-high subgroups based on the optimal cutoff points in the statistical results below. Subsequently, patients were divided into 3 subgroups, namely, RCS 1: both decreased RDW and CEA; RCS 2: decreased RDW with increased CEA or increased RDW with decreased CEA; and RCS 3: both increased RDW and CEA. Other inflammatory prognostic indicators, including the other systematic inflammatory prognostic indicators, including the neutrophil to lymphocyte ratio (NLR), lymphocyte to monocyte ratio (LMR), prognostic nutritional index (PNI), and advanced lung cancer inflammation index (ALI), were determined as described in previous studies [31–33].

### Follow-up and definition of DFS and OS

Patients were routinely followed according to our previous studies [30]. DFS was defined as the date of surgery to the point of any recurrence, metastasis, or death from any cause, and OS was defined from the identical point to the point of any cause of death. The latest follow-up point ended in March 2022.

### Statistical analysis

All analyses were conducted by SPSS 20.0 (SPSS Inc., Chicago, IL, USA) and GraphPad Prism 5 (GraphPad Software Inc., San Diego, CA, USA). The optimal cutoff points of RDW and CEA for the outcome were tested by receiver operating characteristic curve (ROC) analysis. The differences in NLR, LMR, PNI, and ALI in RCS subgroups were analyzed by one-way ANOVA (with LSD for pairwise comparison) or nonparametric tests if a Gaussian distribution was not reached. Survival differences among RCS subgroups were measured by Kaplan–Meier analysis. Risk factors for survival were determined by a Cox proportional hazards model with the iterative forward LR method. *P* < 0.050 was considered statistically significant.

## Results

### Demographic characteristics of the cohort and the efficacy of RCS in prognosis

**Fig. 1:**
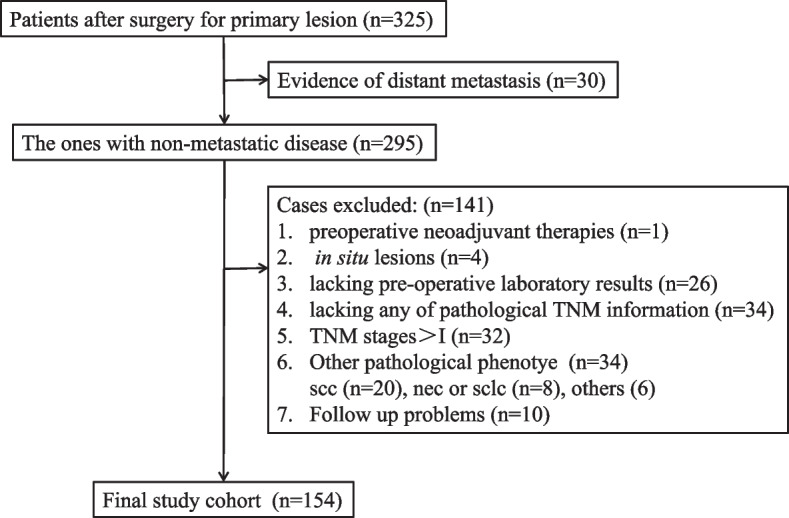
Flow chart of the study. TNM, Tumor-Node-Metastasis; scc, squamous carcinoma; nec, neuroendocrine carcinoma; sclc, small cell lung cancer

**Fig. 2:**
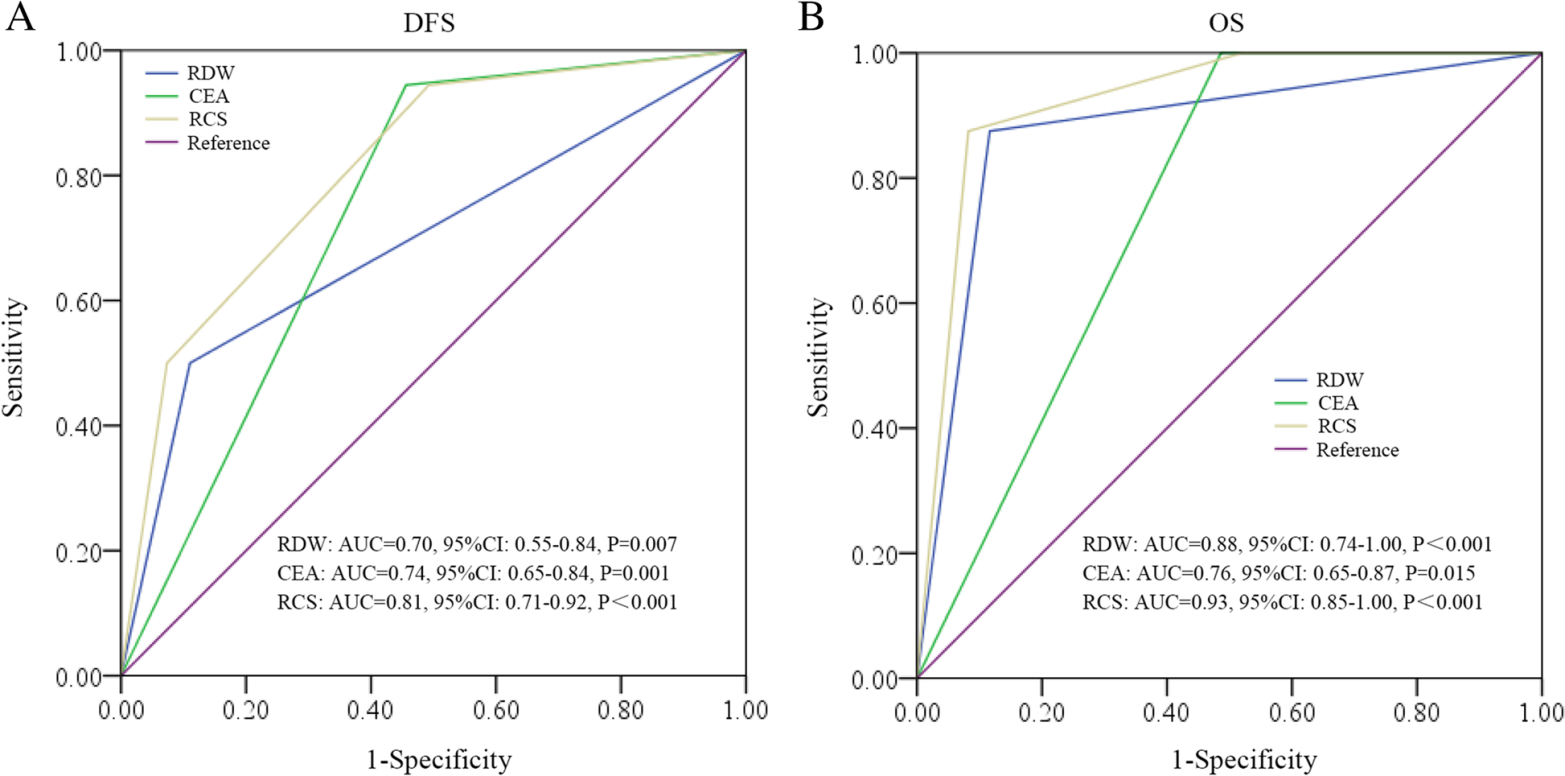
ROC analysis of RDW, CEA, and RCS in predicting DFS (**A**) and OS (**B**). RDW, red cell distribution width; CEA, carcinoembryonic antigen; RCS, red cell distribution width and carcinoembryonic antigen score

In total, 325 patients were included with 30 patients presenting distant lesions; in addition, 141 patients were excluded according to the exclusion criteria (Fig. 1). One hundred fifty-four patients were enrolled in the final cohort, with 124 IA and 30 IB cases. The ratio of female to male was 1:1 (*n* = 77 each), and the median follow-up was 49 months (m) (range: 9–118 m). During the follow-up, 16 patients experienced recurrence, and 8 patients died. By ROC analysis, the RCS was significant in predicting both DFS (AUC = 0.81, *P* < 0.001) and OS (AUC = 0.93, *P* < 0.001), and the AUC was larger than that of RDW or CEA alone (Fig. 2).

### The differences in clinicopathological features among RCS subgroups

**Fig. 3:**
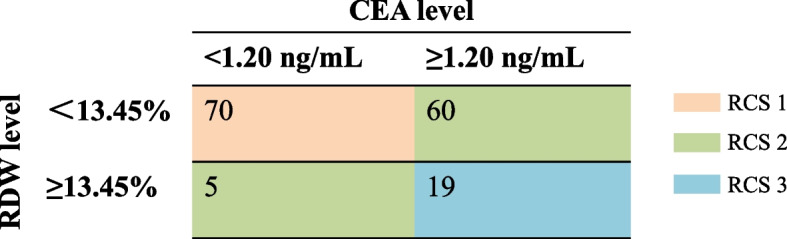
The assignment of patients into different RCS subgroups. RDW, red cell distribution width; CEA, carcinoembryonic antigen; RCS, red cell distribution width and carcinoembryonic antigen score

Taking OS as the end-point, the optimal cutoff points of RDW and CEA to the outcome were determined, and patients were divided into increased (≥ 13.45%) or decreased (< 13.45%) RDW and increased (≥ 1.20 ng/mL) or decreased (< 1.20 ng/mL) CEA subgroups. Based on these results, patients were divided into RCS 1 (*n* = 70, median age: 51 years, range: 29–72 years), RCS 2 (*n* = 65, median age: 60 years, range: 23–79 years), and RCS 3 (*n* = 19, median age: 65 years, range: 49–71 years) subgroups (Fig. 3). It was found that features such as ≥ 60 years (*P* < 0.001), male sex (*P* = 0.004), current or former tobacco use history (*P* = 0.001), T_2_ stage (*P* = 0.004), and IB stage (*P* = 0.006) were more significant in the RCS 2 or 3 subgroups (Table 1).

**Table 1:** Differences of the clinicopathological features in RCS subgroups

**Features**	**Patient No**	**RCS**			
		**RCS 1**	**RCS 2**	**RCS 3**	***P***
**Age (years)**					< 0.001^*^
< 60	90	52	33	5	
≥ 60	64	18	32	14	
**Gender**					0.004^*^
Male	77	25	39	13	
Female	77	45	26	6	
**Type of resection**					0.788
Lobectomy	114	50	50	14	
Segmentectomy	40	20	15	5	
**Micropapillary or solid component**					0.830
Without or unknown	120	56	50	14	
With	34	14	15	5	
**Tobacco use history**					0.001^*^
Never	119	63	45	11	
Current + former	35	7	20	8	
**Alcohol use history**					0.972
Never	100	45	43	12	
Current + former	54	25	22	7	
**Hypertension**					0.060
Without	119	60	45	14	
With	35	10	20	5	
**Combined T stages**					0.004^*^
T_1_	123	63	49	11	
T_2_	31	7	16	8	
**TNM stages**					0.006^*^
IA	124	63	50	11	
IB	30	7	15	8	

^*^With significant statistical difference

*No* Number, *RCS* Red cell distribution width and carcinoembryonic antigen score, *TNM* Tumor-Node-Metastasis

### Correlation of RCS with other systematic inflammatory indicators

**Fig. 4:**
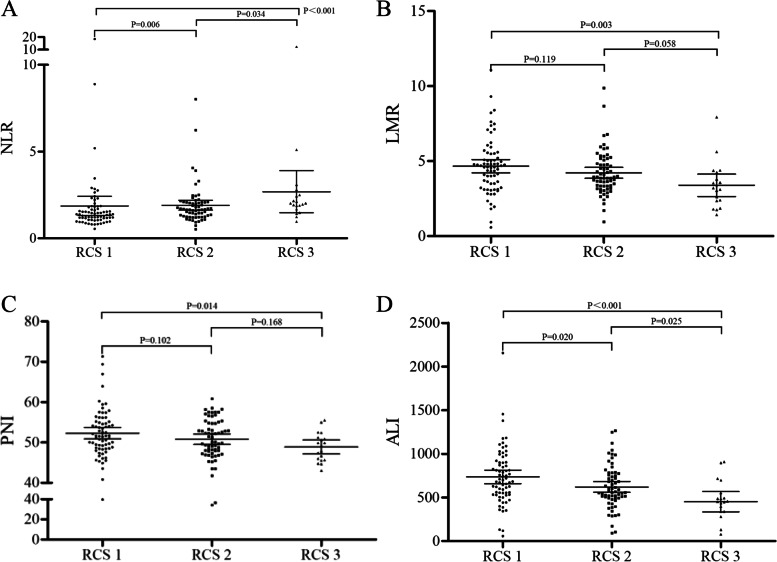
The differences in NLR (**A**), LMR (**B**), PNI (**C**), and ALI (**D**) in the RCS subgroups. NLR, neutrophil to lymphocyte ratio; LMR, lymphocyte to monocyte ratio; PNI, prognostic nutritional index; ALI, advanced lung cancer inflammation index; RCS, red cell distribution width and carcinoembryonic antigen score

As shown in Fig. 4, significant differences were found for NLR, LMR, PNI, and ALI among the different RCS subgroups. In general, a significantly higher NLR was found between the RCS 3 and RCS 2 or 1 subgroups, whereas a significantly lower LMR, PNI, and ALI were also found between the RCS 3 and 1 subgroups and a significantly lower ALI was found between the RCS 3 and 2 subgroups.

### Survival differences among RCS subgroups

**Fig. 5:**
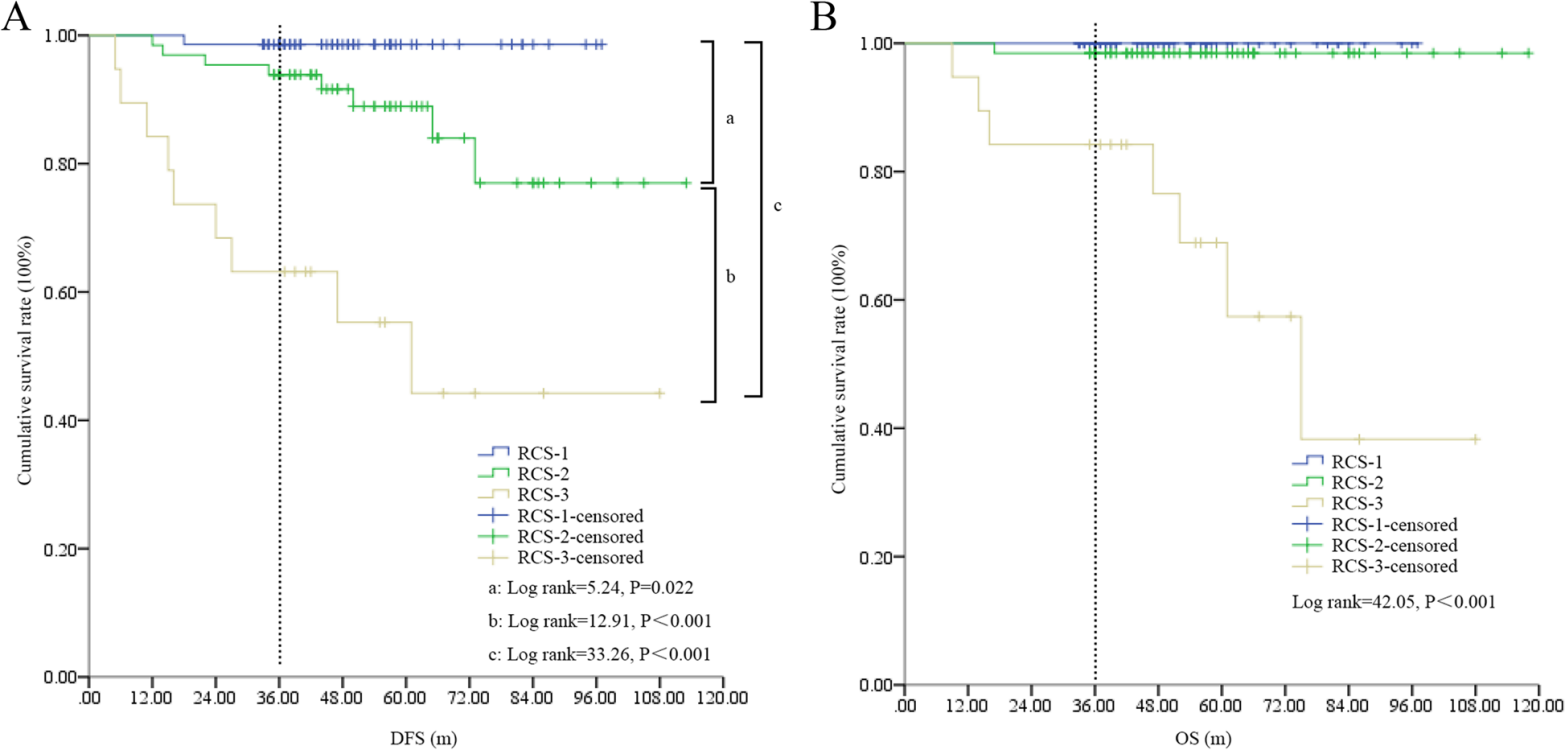
The survival differences among different RCS subgroups. **A** DFS differences in the study cohort. The dotted line indicates 3-year DFS in the subgroups. **B** OS differences in the study cohort. RCS, red cell distribution width and carcinoembryonic antigen score; DFS, disease-free survival; OS, overall survival

By Kaplan–Meier analysis, significant differences in DFS and OS were found among the different RCS subgroups (Fig. 5). Specifically, patients with different RCS could be well separated individually in DFS (the 3-year DFS rates in RCS 1, 2, and 3 were 98.57%, 93.84%, and 63.16%, respectively, *P* < 0.001); however, RCS 2 patients displayed an improved OS, which was similar to RCS 1 patients without a significant difference.

### Univariate and multivariate analysis of risk factors for survival

By the Cox hazard model, with or without micropapillary or solid components, tobacco use history and RCS subgroups were identified as risk factors for both DFS and OS; in addition, with or without hypertension was a risk factor for DFS and TNM stage was a risk factor for OS (Table 2). When these factors were put into multivariate analysis, RCS was found to be an independent risk factor for both DFS and OS (Table 3).

**Table 2:** Univariate tests for risk factors for DFS or OS

	**DFS**			**OS**		
	***P***	**HR**	**95%CI**	***P***	**HR**	**95%CI**
**Age (years)**						
< 60	1			1		
≥ 60	0.097	2.32	0.87–5.76	0.078	4.21	0.85–20.87
**Gender**						
Male	1			1		
Female	0.187	0.52	0.19–1.38	0.215	0.36	0.07–1.80
**Type of resection**						
Lobectomy	1			1		
Segmentectomy	0.635	1.36	0.39–4.77	0.650	1.64	0.19–13.88
**Micropapillary or solid component**						
Without or unknown	1			1		
With	0.014^*^	3.20	1.26–8.13	0.014^*^	6.04	1.44–25.27
**Tobacco use history**						
Never	1			1		
Current + former	0.001^*^	5.10	2.01–12.96	0.010^*^	6.59	1.57–27.68
**Alcohol use history**						
Never	1			1		
Current + former	0.351	1.56	0.61–3.95	0.829	1.17	0.28–4.91
**Hypertension**						
Without	1			1		
With	0.029^*^	2.83	1.12–7.17	0.872	1.14	0.23–5.65
**Combined T stages**						
T_1_	1			1		
T_2_	0.097	2.32	0.87–5.76	0.097	2.32	0.87–5.76
**TNM stages**						
IA	1			1		
IB	0.094	2.32	0.87–6.21	0.027^*^	4.84	1.20–19.58
**RCS subgroups**						
1 + 2	1			1		
3	< 0.001^*^	8.64	3.42–21.79	< 0.001^*^	50.21	6.17–408.60

^*^With significant statistical difference

*DFS* Disease-free survival, *OS* Overall survival, *HR* Hazard ratio, *CI* Confidence interval, *RCS* Red cell distribution width and carcinoembryonic antigen score, *TNM* Tumor-Node-Metastasis

**Table 3:** Multivariate tests for risk factors for DFS or OS

	**DFS**			**OS**		
	**P**	**HR**	**95%CI**	**P**	**HR**	**95%CI**
**Micropapillary or solid component**						
Without or unknown	1			1		
With	0.047^*^	2.69	1.01–7.16	0.035^*^	4.74	1.12–20.11
**Tobacco use history**						
Never	1					
Current + former	0.003^*^	4.44	1.66–11.91			
**RCS subgroups**						
1 + 2	1			1		
3	< 0.001^*^	7.74	2.98–20.08	< 0.001^*^	44.69	5.47–365.29

^*^With significant statistical difference

*DFS* Disease-free survival, *OS* Overall survival, *HR* Hazard ratio, *CI* Confidence interval, *RCS* Red cell distribution width and carcinoembryonic antigen score

## Discussion

In the present study, RCS was found to be a useful prognostic marker in stage I LUAD and could effectively separate the DFS among these patients. RCS 3 patients had worse outcomes than RCS 1 and RCS 2 patients, and it was an independent risk factor for survival. To the best of our knowledge, this is the first study concerning the utility of RCS in lung cancer.

The prognostic value of RDW and CEA has been registered in lung cancer previously. For RDW, Wang et al. conducted a meta-analysis that indicated that pretreatment RDW was significantly associated with poor DFS and OS [10]. However, it was notable that most of the studies enrolled imbalanced stages or mixed pathological phenotypes, and the prognostic value of RDW was likely to be more apparent in early-stage AD cases. For example, Liu et al. conducted a study with 750 stage I–III patients (stage I: *n* = 352, stage II: *n* = 134, stage III: *n* = 264) to explore the prognostic value of RDW, with more than half of the cases being AD (401/750) [34]. Matsui et al. also performed a study with 338 stage I–III patients (stage I: *n* = 289, stage II: *n* = 44, stage III: *n* = 5), and up to 70.38% of the cases were AD (259/338) [35]. To date, only two studies have reported the prognostic value of RDW (226 AD, 47 others [14]; 166 AD [15]) in stage I NSCLC. Moreover, the AUC of RDW alone in predicting survival has been relatively small [11–13]. These results indicated that further improvement of the prognostic efficacy is needed when explored in a specific stage or pathological phenotype. CEA was a useful tumor marker in lung cancer. Interestingly, the prognostic value of CEA in stage I cases has been extensively studied [36, 37]. However, the positive rate of CEA was higher in AD than other pathological phenotypes [24, 25], but similar to RDW, previous studies did not specifically explore its value in AD in stage I cases. In our study, we concurrently explored the prognostic value of RDW and CEA in stage I AD patients, and the results indicated that RCS displayed a larger AUC than RDW and CEA in predicting the outcome. Interestingly, a previous study also investigated the value of a combination of other hematological index (platelet, PLT) with CEA in NSCLC (stage I: *n* = 193, stage II + III: *n* = 83), and the results indicated that the survival was different when patients were divided into PLT^normal^/CEA^normal^, PLT^normal^/CEA^high^, PLT^high^/CEA^normal^, and PLT^high^/CEA^high^ subgroups; however, it was also notable that such algorithm cannot separate the survival effectively in some subgroups [26]. In addition, although not conducted in NSCLC, other studies have indicated that a combination of RDW with tumor markers (CA125 in endometrial cancer [27]; CEA in colorectal cancer [28]) could have better efficacy in prognosis than used individually. Our study finds that RCS could be better in prognostic prediction and could separate the DFS effectively in stage I LUAD; additionally, RCS could be used to identify a cluster of patients with significant inferior OS.

Mechanistically, cancer-related inflammation is thought to be the seventh hallmark of cancer and plays a profound role in cancer initiation and development [38, 39]; in turn, cancer cells can also promote self-development by secreting inflammatory cytokines in an autocrine manner [40]. In lung cancer, some cytokines were significantly elevated in patients, such as interleukin-6 (IL-6) [41, 42], which has broad functions in regulating cancer cells. For example, IL-6 can promote cell proliferation [43], metastasis, and epithelial-mesenchymal transition (EMT) [44]; it can also contribute to treatment (cisplatin [45], EGF receptor (EGFR) tyrosine kinase inhibitors (TKIs) [46]) resistance. Many studies have indicated that abnormally elevated IL-6 predicts poor survival in lung cancer [41, 42, 47]. Interestingly, systematic evaluation of IL-6 can also result in a fluctuation of RDW, as an increase or decrease in RDW was closely correlated with high or low levels of IL-6, although not in the cancer background [48, 49]. In addition, the counts of circulating tumor cells (CTCs) were found to play an essential role in disease recurrence or metastasis in a postoperative setting [50, 51], and these cells were also found to be a major source of CEA in patients [52, 53]. Importantly, some CTCs share the characteristics of cancer stem cells (CSCs) [54, 55], which were identified as the ultimate source of cancer recurrence, metastasis, and treatment resistance [56, 57]. IL-6 can additionally amplify these cells, except for the aforementioned function in cancer cells [43–46]. Based on these findings, RCS 1 patients could mean a decreased concentration of IL-6 and low counts of CTCs or CSCs and also a low mutual promotion between them, which could have a superior outcome than the RCS 2 or 3 patients. Additionally, the prognostic value of other parameters including age [58], gender [58], micropapillary and solid patterns [6], type of resection [58, 59], and smoke and alcohol [60] have been under extensive study in stage I lung cancer, and hypertension was also found contribute to prognosis in lung cancer [61]. It was suggested that male patients smoke in particular after diagnosis correlated with poor survival in stage I cases [58, 60]. In our study, the proportion of male (25/70) and smoke (current + former) (7/70) in the RCS 1 group was significantly lower than in the RCS 2 and 3 subgroups, which may partly support its positive role in survival. Except these, although a high proportion of patients < 60 years was noted in the RCS 1 group in contrast to other subgroups; however, a great number of previous studies indicated that age was not a risk factor for survival in stage I lung cancer patients [62–64]. It was not definite to conclude that age plays a synergic role for good survival in the RCS 1 group at present. Interestingly, we also found a significant difference in other inflammatory prognostic indicators between RCS subgroups, as RCS 1 patients presented the highest NLR and lowest LMR, PNI, and ALI. Although it has not been extensively studied in stage I cases, the low levels of LMR [65], PNI [66], and ALI [67] have been found to be correlated with poor survival in patients after surgery, whereas the high level of NLR was found to be correlated with poor prognosis specifically in stage I cases [68]. These findings also support the notion that RCS 1 patients would have superior survival in our study.

Interestingly, RCS 2 included two subgroups of patients with a reversed increase or decrease in RDW or CEA and displayed a significantly poorer DFS but a similar OS compared with the RCS 1 subgroup. This could also be interpreted from the cancer-related inflammation and CTC perspectives. For DFS, the RCS 2 patients had relatively low levels of IL-6 with a high CTC count or vice versa. For the former, the high count of CTCs was a well-validated poor prognostic indicator in previous studies [50, 51]; for the latter, the CTCs could potentially quickly proliferate with the support of IL-6 [43], both of which could result in an early recurrence or metastasis in contrast to RCS 1 patients. For OS, the subsequent treatment after recurrence or metastasis should be taken into consideration. Notably, more than half of LUAD patients in China harbor EGFR mutations [69, 70], particularly females (66.67% in RCS 2 cases in our study) and never smokers (55.56% in RCS 2 cases in our study) [71, 72], and these patients are commonly treated with TKIs. Notably, it was found that treatment by TKIs such as gefitinib could lead to an obvious decrease in IL-6 in the patients [73]; in addition, such treatment could also shift a portion of patients with high counts of CTCs into low ones, which could have a significantly prolonged PFS [74], which is a surrogate for OS in NSCLC [75]. In addition, we noticed that RCS 3 patients displayed the worst DFS and OS in the cohort, even given that these patients have the same probability of harboring EGFR mutations and undergoing subsequent TKI treatment. We speculate that these patients may have a persistent expansion of CTCs, which could be promoted by a systematic-autocrine IL-6 loop [43] or a shift to the stemness feature of CTCs with IL-6 [76]. Clinically, adjuvant therapies (ADTs) (including chemotherapy, TKIs, or immunotherapy) were still not recommended for stage IA patients and were still controversial in stage IB cases. Based on our results, it was plausible that RCS 1 cases could be waived from ADTs safely due to the outstanding outcome, in addition, although RCS 2 patients were candidates for ADTs due to the relatively poor DFS; however, it was notable that the 3-year absolute benefits from such therapies (taken chemotherapy for example) was only 3.9% and the proportion of 3–4 grade adverse effects was up to 66% [77]; we thus believed “watch and wait” may also be an alternative option for these patients. RCS 3 patients presented the worst DFS and OS in our study, which indicated that these patients were candidates for ADTs and also receive more effective regimens, such as the additional agents anti-IL-6 (ALD518 [78] or siltuximab [79]), in addition to TKIs or TKIs plus chemotherapy based on our aforementioned speculation.

There are certain limitations to the present study. First, it was a retrospective study performed in a single hospital, and the sample size was relatively limited; in particular, only 19 patients were assigned to the RCS 3 group, which could bias the results. Second, the information of gene testing and the subsequent therapy regimens were not definite in our cohort, which therefore could not support our speculation. Some evidence for our speculation could be obtained if post hoc analysis could be conducted like the LACE study [77] where RCS could be analyzed as a stratification factor. Third, as pure solid type could have different CTCs in NSCLC [80], we could not analyze the pathological phenotype to the prognosis since 30 patients did not report definite pathological elements in resected samples. Fourth, taking into account the fact that RDW and CEA could be easily measured during subsequent treatment or follow-up, repeated assessments to validate its prognostic value in stage I LUAD are achievable. Nonetheless, more studies with a large sample size, in particular the randomized controlled trials, are the best way to validate our findings in the future.

## Conclusion

Overall, our study indicated that RCS was a useful prognostic marker in stage I LUAD patients and RCS 3 patients had the worst survival. In addition, taking into account the fact that the poor OS in RCS 3 patients, we speculate that more effective ADTs should be an option for these patients; however, randomized controlled trials are needed in the future.

## Data Availability

The datasets generated or analyzed during the current study are available from the corresponding author on reasonable request.
